# The Expanded Regulatory Significance of Saharan Dust Plumes in the United States

**DOI:** 10.1021/acs.est.5c02205

**Published:** 2025-09-08

**Authors:** Paul Miller, Kimberly Hamilton-Wims, Ken Holmes, Doug Melancon, Jason Meyers, Tegan Treadaway

**Affiliations:** † Coastal Meteorology (COMET) Lab, Department of Oceanography and Coastal Sciences, Louisiana State University, 93 S Quad Dr., Baton Rouge, Louisiana 70803, United States; ‡ Coastal Studies Institute, Louisiana State University, 93 S Quad Dr., Baton Rouge, Louisiana 70803, United States; § Office of Environmental Assessment, Louisiana Department of Environmental Quality, 602 N Fifth St., Baton Rouge, Louisiana 70802, United States; ∥ Baton Rouge Complex, ExxonMobil, Baton Rouge, 5955 Scenic Hwy, Louisiana 70805, United States

**Keywords:** Saharan dust, air quality, particulate matter, annual PM_2.5_ NAAQS, regulation

## Abstract

Given the recent reduction in the U.S. National Ambient Air Quality Standard (NAAQS) for annual PM_2.5_ from 12 to 9 μg m^–3^, the contribution of exceptional, though natural, particulate transport events has assumed greater regulatory relevance. This study examines the evolution of a June 2022 trans-Atlantic Saharan dust outbreak and contextualizes its impact on the 2023 PM_2.5_ design values (DVs) for continental U.S. air quality monitors. The results demonstrate that the intrusion of Saharan dust yielded mean 24 h PM_2.5_ values > 35 μg m^–3^ for multiple days across the Central U.S. and single-handedly elevated the 2022 mean annual PM_2.5_ by > 0.5 μg m^–3^ for some Gulf Coast communities. Moreover, the Saharan dust outbreak led to ∼0.1 μg m^–3^ increases in the 2023 PM_2.5_ DVs for approximately 20 monitoring sites in Texas and Louisiana, and in the case of Port Allen, La., and at least two other monitors, the Saharan dust pushed the DV above the 9 μg m^–3^ NAAQS. As this June 2022 episode illustrates, seemingly rare atmospheric phenomena are positioned to play disproportionately large regulatory roles, given the tighter air policy landscape.

## Graphical abstract

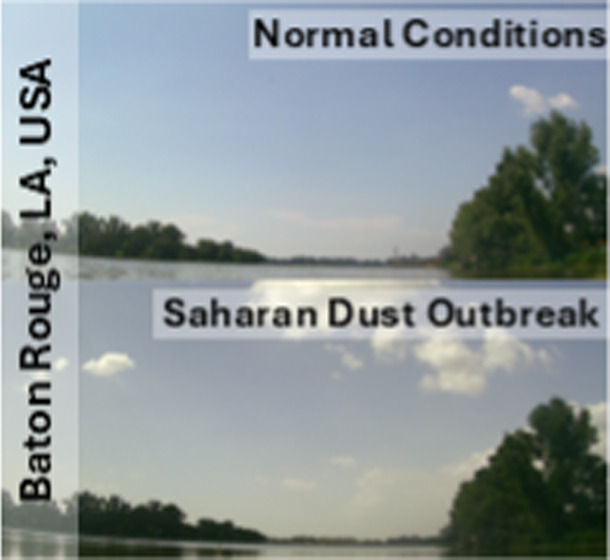

## 1 Introduction

The Saharan air layer (SAL) is an endemic feature of the tropical North Atlantic. This hot, dry air mass is laden with dust aerosols that are mobilized over the Sahara and zonally transported into the Atlantic by the easterly trade-wind regime.^1^ The Sahara is estimated to mobilize ∼2100 Tg of dust emissions each year,^2^ and 72 Tg are advected beyond 15°W toward the Americas.^3^ However, between late May and early August, partially coinciding with Atlantic hurricane season,^4^ the SAL flares much further across the Atlantic, sometimes reaching the eastern Caribbean^5^ within 5–7 days.^6^ Approximately 18 Tg of dust reaches 75°W at the latitude of the Caribbean basin.^3,7^ In some cases, SALs may even penetrate all the way into the Gulf of America and impact locations in the central and eastern U.S.^8^

The SAL has been shown to produce myriad environmental impacts across the Earth system. For instance, mineral dust serves as an important nutrient source, particularly iron, for the tropical Atlantic Ocean,^9,10^ and similarly, SAL-transported aerosols play an important role in nutrient cycling of the Amazon rainforest.^11^ Meanwhile, the hot, dry, desert-origin air pocket encapsulating the dust stifles embryonic thunderstorm activity in the Caribbean,^12^ leading to diminished rainfall and in some circumstances, even drought.^13^ In addition to water resources strain, Saharan dust also diminishes visibility in its destination regions,^14^ with SAL encounters often associated with haze reports in local weather bulletins,^15^ and affects cloud formation in dust-prone regions.^16,17^

Additionally, the SAL poses a human health risk to populations along Atlantic coastlines.^18,19^ Saharan dust is a form of fine- and coarse-mode particulate matter (PM), defined as aerosols with diameters of ≤2.5 μm (i.e., PM_2.5_) and ≤10 μm (i.e., PM_10_), respectively. The SAL’s high PM load can cause respiratory harm to vulnerable populations, such as asthmatics and children.^20^ For instance, in Puerto Rico, 90% of survey respondents indicated that Saharan dust impacted the health status of either themselves or their family members.^21^ Similarly, PM_10_ related to African dust events, particularly those impacting urban centers in Puerto Rico, were associated with greater proinflammatory responses in the human respiratory system.^22^

Beyond circumstantial human health interests, the SAL’s air-quality impacts, particularly for PM_2.5_, have recently assumed a significant regulatory relevance. In February 2024, the U.S. Environmental Protection Agency (EPA) revised the National Ambient Air Quality Standard (NAAQS) for annual PM_2.5_ concentration to 9 μg m^–3^ from its previous standard of 12 μg m^−3^.^23,24^ The PM_2.5_ design value (DV), used to determine compliance with the NAAQS, is produced by computing the annual mean PM_2.5_ value from all daily means available during that year for a monitor. The annual means of the most recent three years of complete data are then averaged to yield the DV for the monitor, with the final year being used to label the DV. For instance, the 2020 DV would have been computed by separately averaging all available daily values for 2018, 2019, and 2020 to obtain three annual means, which are then themselves averaged to yield the DV. At the time of the annual PM_2.5_ NAAQS revision, the most recent three-year period available for designating attainment versus nonattainment was 2021–2023 (i.e., the 2023 DV). Figure 1a shows the distribution of 2023 DVs for the U.S., which includes dozens of locations that satisfied the old NAAQS but now face apparent nonattainment of the revised standard (red dots). Additionally, many other monitoring stations are now within 1 μg m^–3^ of nonattainment, which can influence future emissions permitting and future attainment.

**Figure 1:**
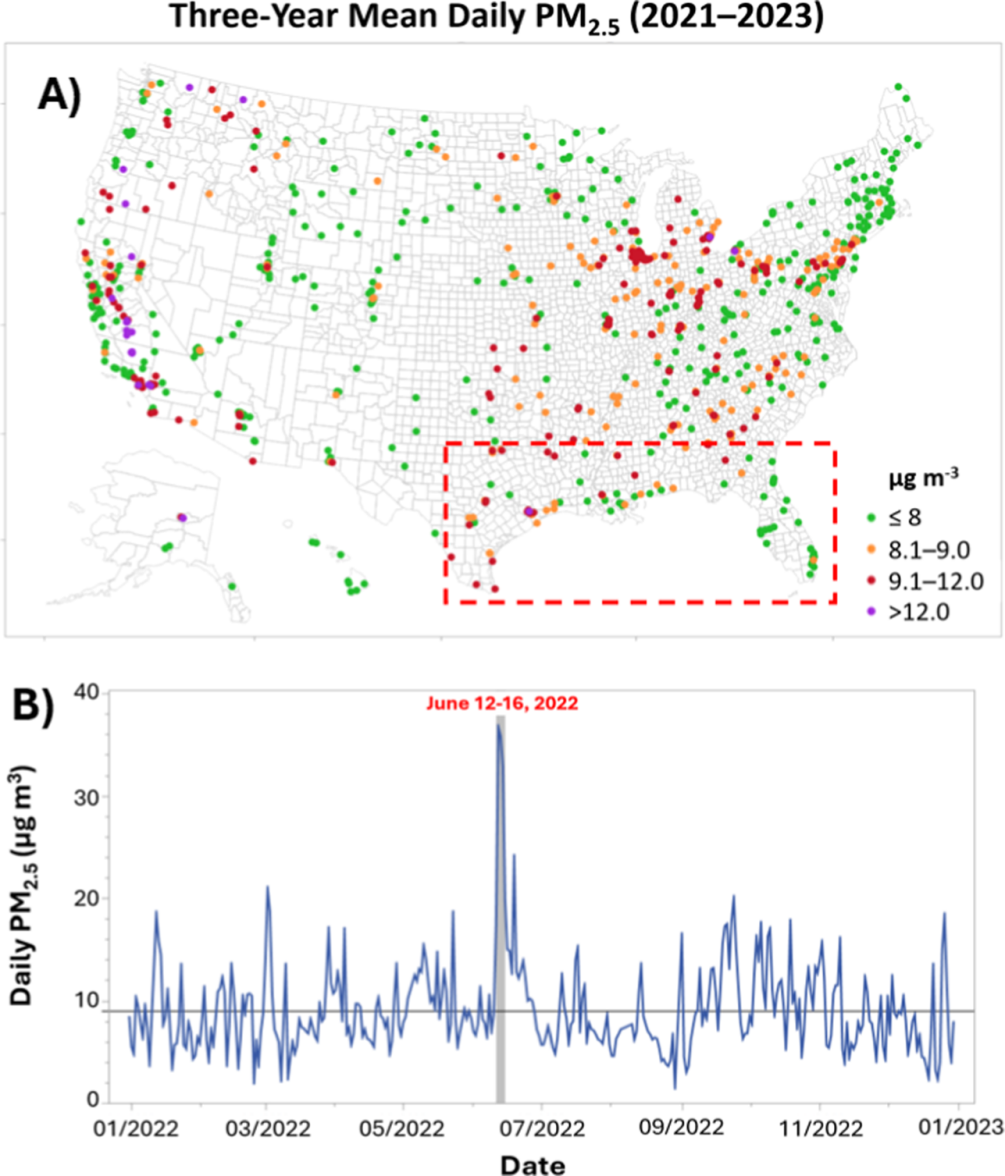
(A) PM_2.5_ design values (μg m^–3^) for 2023 computed from annual averages between 2021–2023 for the 803 qualifying monitors. Air monitors are binned as follows: ≤8 (*n* = 424; 53%), 8.1–9.0 (*n* = 200; 25%), 9.1–12.0 (*n* = 156; 19%), and ≥12 μg m^–3^ (*n* = 23; 3%). The dashed red box captures the northern Gulf of America (80–100°W; 23–33°N). (B) Time series of daily mean PM_2.5_ values (μg m^–3^) at the Port Allen monitor during 2022. The period between June 12 and 16 is shaded gray. The black line corresponds to 9 μg m^–3^.

Because SAL events easily prompt daily PM_2.5_ to exceed 30 μg m^–3^ (and may exceed 50 μg m^–3^ in some cases) for several days on end,^25^ SAL intrusions among EPA jurisdictions now possess significant regulatory importance, particularly for industrialized locations in the U.S. Caribbean and Gulf Coasts,^26^ where Saharan dust outbreaks can confound the annual PM_2.5_ DV.^27^ The purpose of this study is to highlight the regulatory significance of a Saharan dust outbreak during June 2022 as an archetype for such events. While a primary focus of SAL-related research has been on its physical role within the earth system,^2^ the recent revision to the annual PM_2.5_ NAAQS will require scientists and regulators to place a greater emphasis on Saharan dust’s air quality implications. Though analyses like those conducted herein can also be gleaned from obscure EPA filings and statutorily mandated public-comment portals, this article also intends to introduce the newfound air-quality considerations of SAL events to the broader scientific community.

## 2 Data and Methods

The June 2022 SAL event was detected by several satellite systems and was well resolved by aerosol-enabled numerical modeling systems. This study will leverage true-color satellite imagery from Terra’s MODIS sensor and numerically computed fields from NASA’s Modern-Era Retrospective Analysis for Research and Applications, Version 2 (MERRA-2), reanalysis data set. MERRA-2 reanalysis is widely used and trusted for particulate transport analyses,^28–30^ given its sophisticated atmospheric chemistry parameterizations, which isolate individual particulate species and sizes—including dust <2.5 μm.^31^ The five aerosol species in MERRA-2 (sea salt, sulfate, black carbon, organic carbon, and dust) can also be combined to yield the overall PM_2.5_ concentration.^32,33^

In-situ surface observations are retrieved from the EPA’s daily PM_2.5_ FRM/FEM Mass (88101) archive,^34^ AirNow portal,^35^ and PM_2.5_ Tiering Tool.^36^ Only 24 h bulk averages are considered because they represent the most common form of daily frequency measurement in the FRM/FEM data set and will serve as the basis of the DV computation. Because the DVs computed herein may not match EPA official values in all cases, they should be interpreted as pseudovalues. The daily PM_2.5_ values as well as the associated air quality index (AQI) are plotted in conjunction with the MERRA-2 fine particulate fields to demonstrate the correspondence between modeled aerosol transport and observed air quality deterioration. The AQI is a unitless value computed by interpolating each criteria pollutant’s concentration between prescribed pollutant-specific categories of ascending health concern.^37^ The resultant index is assigned to one of six categories that qualitatively communicate the current or forecast air quality conditions to the broader public. The AQI ranges and categorical labels utilized by the EPA are as follows: Good (AQI 0–50; PM_2.5_ ≤ 9.0 μg m^–3^), Moderate (AQI 51–100; PM_2.5_ 9.1–35.4 μg m^–3^), Unhealthy for Sensitive Groups (AQI 101–150; PM_2.5_ 35.5–55.4 μg m^–3^), Unhealthy (AQI 151–200; PM_2.5_ 55.5–125.4 μg m^–3^), Very Unhealthy (AQI 201–300; PM_2.5_ 125.5–225.4 μg m^–3^), and Hazardous (AQI 301+; PM_2.5_ ≥ 225.5 μg m^–3^).

## 3 Results

This analysis will first document the synoptic journey and air quality impacts of the June 2022 SAL incursion over the continental U.S., before more closely examining its site-specific role in an apparent NAAQS exceedance in Port Allen, La., and two other monitors. Though these locations did not experience the most severe PM_2.5_ concentrations, the Saharan dust outbreak combined with the local ambient particulate levels to yield apparent nonattainment of the revised annual PM_2.5_ NAAQS. The analysis will first examine observed surface PM_2.5_ (Section 3.1) and trace the origin of the high PM_2.5_ eastward toward the Sahara (Section 3.2). The high PM_2.5_ levels will then be contextualized within the 2023 design values of air quality monitors in the continental U.S. (Section 3.3), and the regulatory significance of dust among Gulf-adjacent monitors will be presented as an archetype of future events (Section 3.5).

### 3.1 Surface PM_2.5_ Observations

The 2022 time series of daily PM_2.5_ at the Port Allen monitor is shown in Figure 1b, with the June 12–16 period representing the highest concentrations of the year by a significant margin. Table 1 contains measured PM_2.5_ concentrations from Port Allen and three additional monitoring sites along the U.S Gulf Coast during mid-June 2022, where these heaviest dust concentrations occurred, many of which exceeded the 99th percentile for the monitor’s entire period of web archival. The two easternmost sites (Port Arthur and Port Allen) experienced their greatest PM_2.5_ exposures on June 13, whereas the monitors in Corpus Christi and Houston encountered two peaks in surface PM_2.5_. In Corpus Christi, PM_2.5_ neared 50 μg m^–3^ on two occasions, June 12 and June 16, while Houston’s concentrations were roughly 10 μg m^–3^ lower. Figure S1a,b shows the visible haze created by the high dust concentrations in Baton Rouge, LA, a few miles east of the Port Allen monitor on June 14.

**Table 1:** Daily PM_2.5_ Values (μg m^–3^) during the 2022 Dust Episode for Select Locations in the U.S. Gulf Coast, including Kingsville, Texas (AQS 482730314; avail.: 2003-2024), Houston, Texas (AQS 482010046; avail.: 2021-2024), Port Arthur, Texas (AQS 482450021; avail.: 2000-2024), and Port Allen, La. (AQS 221210001; avail.: 1999-2024)^a^

Date	Kingsville, TX	Houston, TX	Port Arthur, TX	Port Allen, LA
12 June	48.5 (99.9)	30.9 (99.1)	32.2 (99.5)	17.5 (87.0)
13 June	36.4 (99.7)	33.9 (99.6)	38.8 (99.8)	36.7 (99.8)
14 June	29.8 (98.9)	28.6 (98.5)	28.7 (99.2)	35.8 (99.7)
15 June	38.5 (99.8)	24.0 (94.6)	21.7 (96.8)	32.9 (99.5)
16 June	46.0 (99.9)	38.8 (99.7)	23.7 (97.8)	19.8 (91.2)

a The percentile of the daily value within the monitor’s period of web archival is provided parenthetically. The nearest city and monitoring station identification number within the EPA Air Quality System (AQS) is provided. The 2023 PM_2.5_ DVs for the four sites noted below are 9.9, 12.5, 8.8, and 9.1 μg m^–3^, respectively. The location of the Port Allen monitor is denoted in Figure 2.

### 3.2 Path of the June 2022 Dust Episode

The origins of the high PM_2.5_ concentrations presented in Section 3.1 are traced to the eastern Atlantic more than a week beforehand when a mass of Saharan dust was ejected off the African Coast in early June (Figure S1c). In addition to the visible satellite images of the plume’s origin, the dust mass was also well resolved by the MERRA-2 reanalysis data set. Figure S1d shows the 24 h mean dust-only PM_2.5_ concentration, which closely matches the visible satellite image in Figure S1c. Figure 2a–c shows the movement of the dust layer in 48 h increments between June 7–11, 2022, as it traverses the tropical North Atlantic, following a well-documented Saharan dust transport pathway during boreal summer.^38,39^ Though Saharan dust plumes can often be fortified by aerosol species from additional sources, such as carbonaceous aerosols from biomass burnings,^40^ Figures S2 and S3 demonstrate that the aerosol plume shown in Figure 2 is overwhelmingly composed of dust.

**Figure 2:**
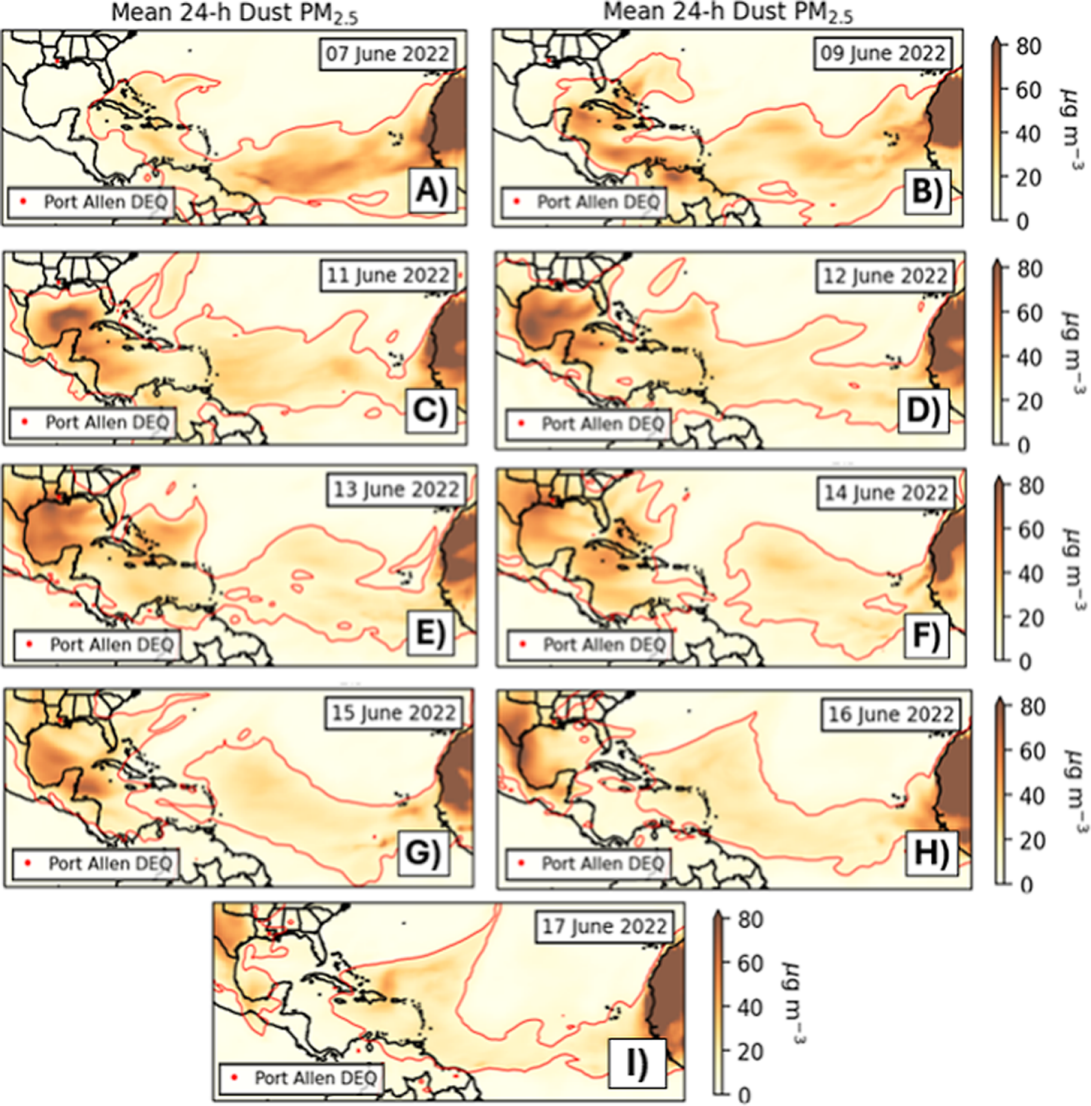
MERRA-2 dust-only PM_2.5_ concentration for the 24 h period beginning 0000 UTC daily on (A) June 7, 2022, and continuing for (B–C) June 9 and 11 and (D–I) June 12–17, 2022. The Port Allen monitor is shown in a red circle, and the 9 μg m^–3^ contour is drawn in a red line. See Figure S4 for annotations denoting relevant meteorological features.

On June 7, easterly trade winds associated with anticyclonic circulation around the North Atlantic Subtropical High (NASH) direct the plume westward (Figures 2a and S4a), and on June 9, the dust plume, continuing its westward migration (Figure S4b), enters the Caribbean basin (Figure 2b). Meanwhile, a low-pressure system along the eastern U.S. seaboard erodes the western edge of the NASH (Figure S4b), allowing the dust to advect further north into the Greater Antilles, the Bahamas, and South Florida. Two days later, the Saharan dust pulse rounds the western flank of NASH (Figure S4c) and is firmly located within the Gulf of America (Figure 2c).

On June 12, the stationary front along the Gulf Coast lifts poleward as a warm front (Figure S4d), allowing the dust mass to move northward into the continental U.S. On the following day, June 13, 2022, the western Gulf Coast is firmly embedded within the Saharan dust plume (Figure 2e). Southerly surface flow characterizes the entire Southern Plains and Lower Mississippi River Valley, continuing equatorward into the Gulf of America (Figure S4e). This wind pattern creates a continuous pathway for the advection of the Saharan dust plume into the Gulf Coast and Southern Plains. While MERRA-2 resolves 24 h mean dust PM_2.5_ in the 30–40 μg m^–3^ range, even larger values remain offshore (Figure 2e). The MERRA-2 PM_2.5_ concentrations match the >30 μg m^–3^ observed PM_2.5_ values from June 13, 2022 (Table 1).

The incipient low-pressure center shown in Figure S4e has now deepened into a 991 hPa cyclone with attendant cold and warm fronts (Figure S4f). This classical midlatitude cyclone structure continues to place Texas and Louisiana in the region of southerly surface winds, advecting Saharan dust into the northern Gulf Coast for the third consecutive day (Figure 2f). MERRA-2 resolves a 24 h mean dust PM_2.5_ of ∼40 μg m^–3^, which corresponds well to the 35.8 μg m^–3^ observed at Port Allen on June 14 (Table 1). Southerly warm-sector surface winds continued on June 15, 2022 (Figure S4g), aided by the development of a 1018 hPa high over the central Gulf of America. However, the dust intensity begins to abate as 3 days of poleward migration have dispersed the particulates over a significant area of the continental U.S. A secondary lobe of Saharan dust is also now apparent, stretching from the northwest Caribbean to the western Gulf of America (Figure 2g).

On June 16, 2022, the 1018 hPa anticyclone from Figure S4g has translated westward so that it now lies directly offshore of the Louisiana coast (Figure S4h), disrupting the persistent southerly flow that has advected dust into the Central U.S. Simultaneously, the 1018 hPa anticyclone pushes the secondary dust lobe further westward onto the Texas coast, preventing the subsequent dust pulse from impacting Louisiana (Figure S4h). On June 17, 2022, the 1016 hPa isobar has expanded from its position on June 15–16, 2022, while the 1018 hPa high-pressure center has marginally strengthened to 1019 hPa and moved over the southern Mississippi (Figure S4i). The expansion of higher pressure from the east ushers in nondust-laden air from the Southwest Atlantic (Figure 2i), which finally leads to the reduction of dust PM_2.5_ below 9 μg m^–3^ across most of the Gulf of America.

### 3.3 Impacts of Dust on Continental U.S. Air Quality

Figure 3 demonstrates the superposition of numerically modeled dust transport and observed reductions in air quality using the AQI category as a qualitative description of air quality. Upon approaching the U.S. coastline, the Saharan dust plume leads to more severe AQI categories, indicating the deterioration of local air quality in association with the dust’s arrival. On June 11, while the SAL outbreak is still offshore, nearly every air quality sensor in the continental U.S. reports an AQI in the “good” range (Figure 3a). However, as the Saharan dust front crossed the Gulf Coast, the AQI increased to “moderate” for effectively all stations within the 9 μg m^–3^ contour (Figure 3b). Additionally, dozens of monitors in the Ohio River Valley report a “moderate” AQI outside of the 9 μg m^–3^ contour on June 13 (Figure 3c), reinforcing the notion that dust PM_2.5_ can exacerbate what would otherwise be only marginally high ambient PM_2.5_. Meanwhile, approximately six monitoring stations in Louisiana and Texas report an AQI in the “unhealthy for sensitive groups” range on this day.

**Figure 3:**
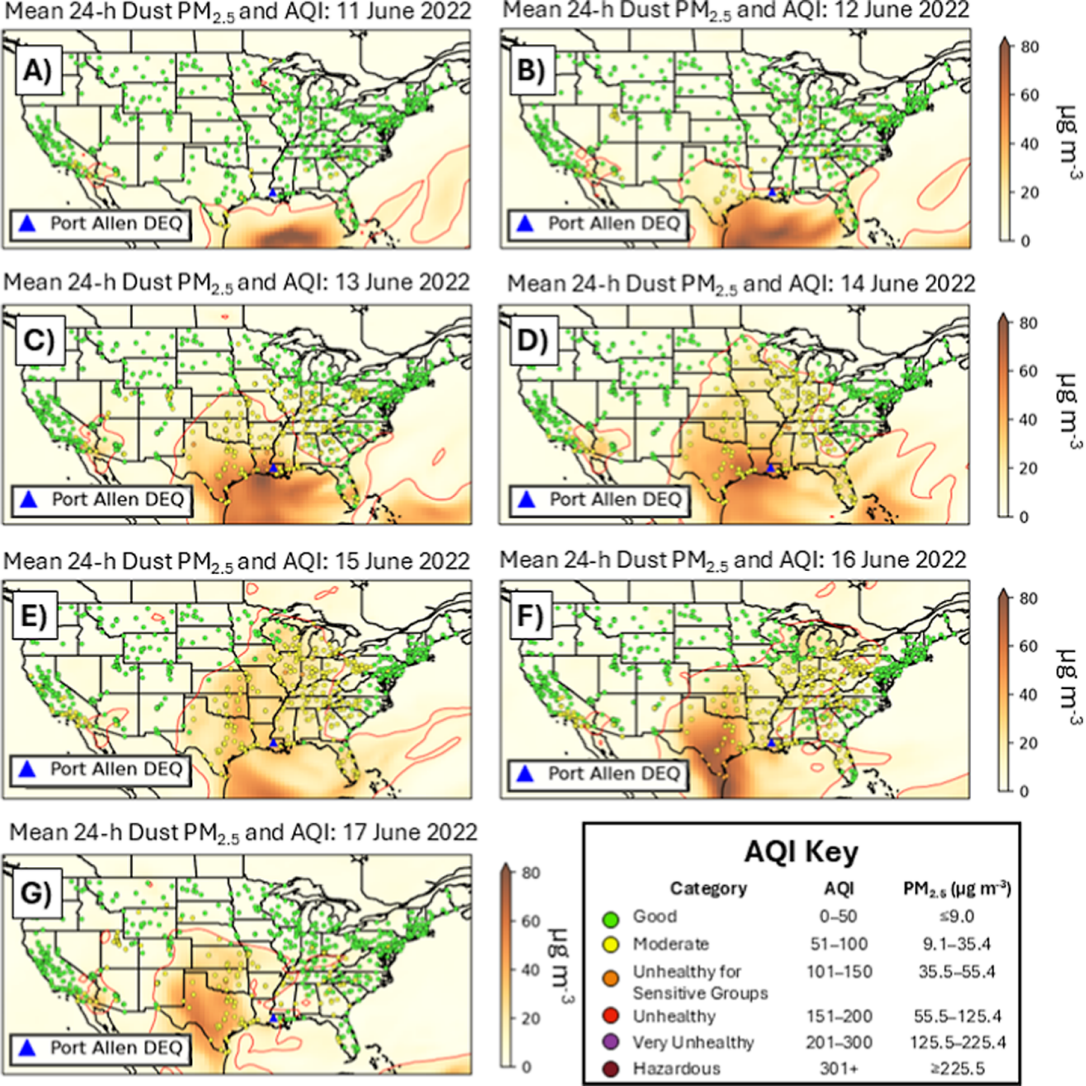
Evolution of daily mean PM_2.5_ values (μg m^–3^) from June 11 to 17, 2022, in comparison to the observed particulate AQI at FRM/FEM monitoring stations across the country. The 9 μg m^–3^ contour is drawn in red.

During June 14–15, 2022, the middle third of the continental U.S. is characterized by “moderate” AQIs, almost identically corresponding to the footprint of the 9 μg m^–3^ dust-only contour (Figure 3d,e). While PM_2.5_ decreases across the Midwest and Great Lakes according to the MERRA-2 reanalysis, these regions continue to report “moderate” AQIs for a third day (Figure 3f). Simultaneously, the secondary pulse of Saharan dust mentioned in Figure 2g generates a localized region of “unhealthy for sensitive groups” along the U.S.–Mexico border, corresponding to the latter PM_2.5_ maxima shown in Table 1. By June 17 (Figure 3g), this second thrust of Saharan dust had begun to disperse, and the daily AQI across the continental U.S. had improved to its healthiest levels since the first intrusion of Saharan dust on June 12 (Figure 3b).

Whereas Figure 3 examines the nationwide evolution of the daily AQI in relation to the arrival and dissipation of the Saharan dust plume, Figure 4 contextualizes these dust levels relative to the monitors’ annual mean PM_2.5_ as well as their associated DVs (i.e., the mean of three annual averages for a rolling three-year period; see Section 1). Figure 4a shows that the maximum observed daily PM_2.5_ during this five-day window exceeded 30 μg m^–3^ from the U.S.–Mexico border all the way to the Great Lakes region. More broadly, nearly the entire eastern U.S., aside from New England, experiences some form of elevated PM_2.5_ (i.e., >10 μg m^–3^) during this period. Figure 4b translates the contribution of the June 12–16 SAL episode to the annual mean PM_2.5_. While a limited number of monitoring stations near the U.S.–Mexico border experienced annual increases >0.40 μg m^–3^, most locations in Texas and Louisiana witness mean PM_2.5_ boosts between 0.2 and 0.4 μg m^–3^. In terms of the 2023 DV, which determines the attainment versus nonattainment classification relative to the revised annual PM_2.5_ NAAQS, the 5 days between June 12 and 16, 2022, represent only 0.46% of the DV period, but they singlehandedly boosted the 2023 DV by 0.1 μg m^–3^ at 24 monitor locations. Meanwhile, this Saharan dust outbreak served to increase the 2023 DV for 83.4% of the monitors pictured in Figure 4c. This is particularly notable because 2022 was an otherwise low-PM_2.5_ year within the 2023 DV period (i.e., 2021–2023), representing the lowest mean annual PM_2.5_ of the three-year period for 63.6% of qualifying monitors nationwide (600/943 = 63.6%). Given that days-long Saharan dust intrusions can elevate DVs by up to 0.1 μg m^–3^ (Figure 4c), Figure 4d visualizes the locations across the continental U.S. where 2023 DVs exceeded the annual PM_2.5_ NAAQS by < 0.1 μg m^–3^. In summary, Figure 4 illustrates the locations where Saharan dust intrusions could plausibly create regulatorily significant PM_2.5_ increases in light of the revised annual NAAQS.

**Figure 4:**
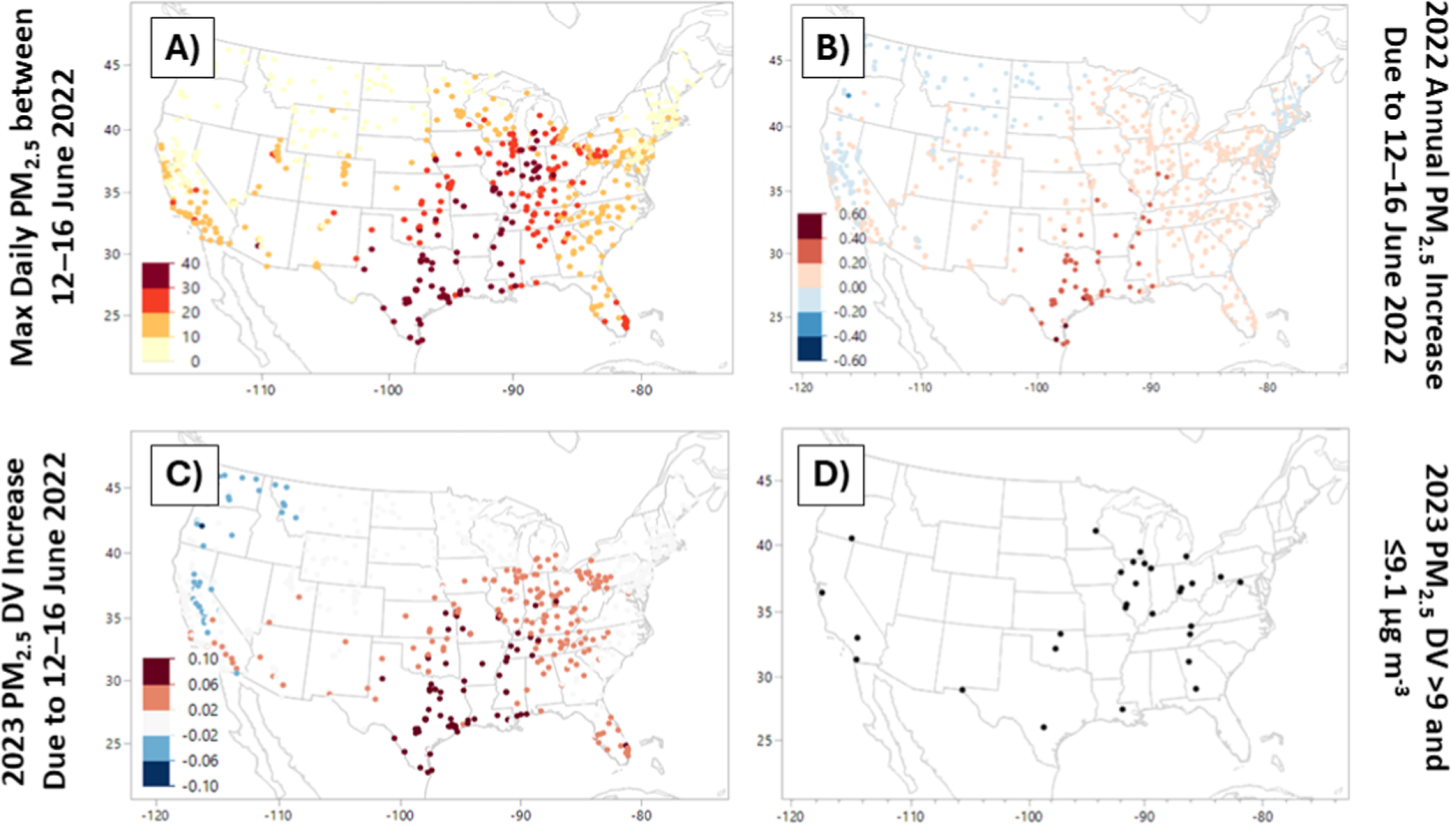
(A) Distribution of maximum 24 h PM_2.5_ during the Saharan dust outbreak, as well as (B) the change in the 2022 annual mean PM_2.5_ due to this five-day episode. (C) The change in the 2023 DV due to this same five-day period. (D) The locations of any PM_2.5_ monitors whose 2023 DVs were within 0.1 μg m^–3^ of the revised PM_2.5_ annual NAAQS, indicating locations where similar Saharan dust episodes may pose regulatory significance.

### 3.4 Monitor-Scale Case Study

Special attention is paid to the Port Allen, La., monitor (AQS ID: 221210001) because of its unique regulatory position, which can be demonstrated by Table 1 and Figure 4. Port Allen experienced prominent impacts from the Saharan dust outbreak and also reported a 2023 DV slightly above the 9 μg m^–3^ annual PM_2.5_ NAAQS (Figures 1a and 4d). Thus, it represents an archetype that may become increasingly common: The encroachment of Saharan dust into the continental U.S. yields regulatorily significant changes to formal air quality compliance designations (i.e., attainment versus nonattainment). The daily PM_2.5_ during the June 12–16, 2022, dust episode is nearly double the next highest daily PM_2.5_ during the three-year window, and the peak of the dust PM_2.5_ measured on June 13 (36.7 μg m^–3^; Table 1) is also the largest value measured during all of 2022 by a significant margin. Moreover, the air mass prior to arrival of the dust was relatively “clean” with PM_2.5_ < 10 μg m^–3^, which quadruples upon the arrival of the dust shown in Figures 1a, 2, and 3.

Table 2 shows the impact of including versus excluding this five-day period of significant Saharan dust activity within the 2023 DV. Prior to the revision of the NAAQS for annual PM_2.5_, Port Allen would have satisfied the attainment criterion (i.e., 12 μg m^–3^) regardless of the SAL’s presence. However, following the tightening of the standard, the Saharan dust outbreak represents a critical tipping point for the monitor. If included in the 2023 DV, then Port Allen would be designated as “nonattainment” by the U.S. EPA. However, if the June 12–16 Saharan dust episode is excluded from the DV calculation, Port Allen would satisfy the NAAQS and be designated as “attainment.” Because nonattainment designation results in a slew of new and costly regulations for facilities in the nonattaining area,^41^ the exclusion of the June 2022 SAL event from the DV calculation possesses immense economic consequences. Table 2 also demonstrates similar situations in both Evansville, Ind., and Oklahoma City, Okla.

**Table 2:** Annual PM_2.5_ (μg m^–3^) Averaged for the Port Allen, La. (AQS 221210001), Evansville, Ind. (AQS 181630016), and Oklahoma City, Okla. (AQS 401090097), Monitoring Stations, Both Including and Excluding the Five Daily Values from June 12 to 16, 2022^a^

Year	Port Allen, La.		Evansville, Ind.		Oklahoma City, Okla.	
	included	excluded	included	excluded	included	excluded
2021	8.76 (352)	8.76 (352)	9.51 (118)	9.51 (118)	10.07 (334)	10.07 (334)
2022	9.85 (354)	9.13 (349)	8.79 (116)	8.60 (114)	8.24 (352)	8.09 (347)
2023	9.32 (342)	9.32 (342)	8.86 (117)	8.86 (117)	8.79 (332)	8.79 (332)
DV	9.07	8.98	9.05	8.99	9.04	8.98

a The number of daily observations contributing to the yearly averages is shown parenthetically.

Beyond the ramifications in Table 2, dust effects can combine with other natural events to cumulatively yield regulatorily significant situations elsewhere. For instance, expansive Canadian wildfire activity during May–June 2023 elevated eastern-U.S. PM_2.5_ and ozone concentrations to unprecedented levels.^42^ Thus, some air monitors whose 2023 DVs were slightly above the new 9 μg m^–3^ NAAQS (Figure 5d) may find that the dust is regulatorily significant when analyzed in conjunction with other natural, yet exceptional, particulate-producing events. Even in the absence of outright changes in “attainment” designation, the dust-related DV elevation in Figure 4c represents, at a minimum, a loss of “headroom”,^43^ whereby industrial activities face greater permitting scrutiny when the DV is close to the NAAQS.

**Figure 5:**
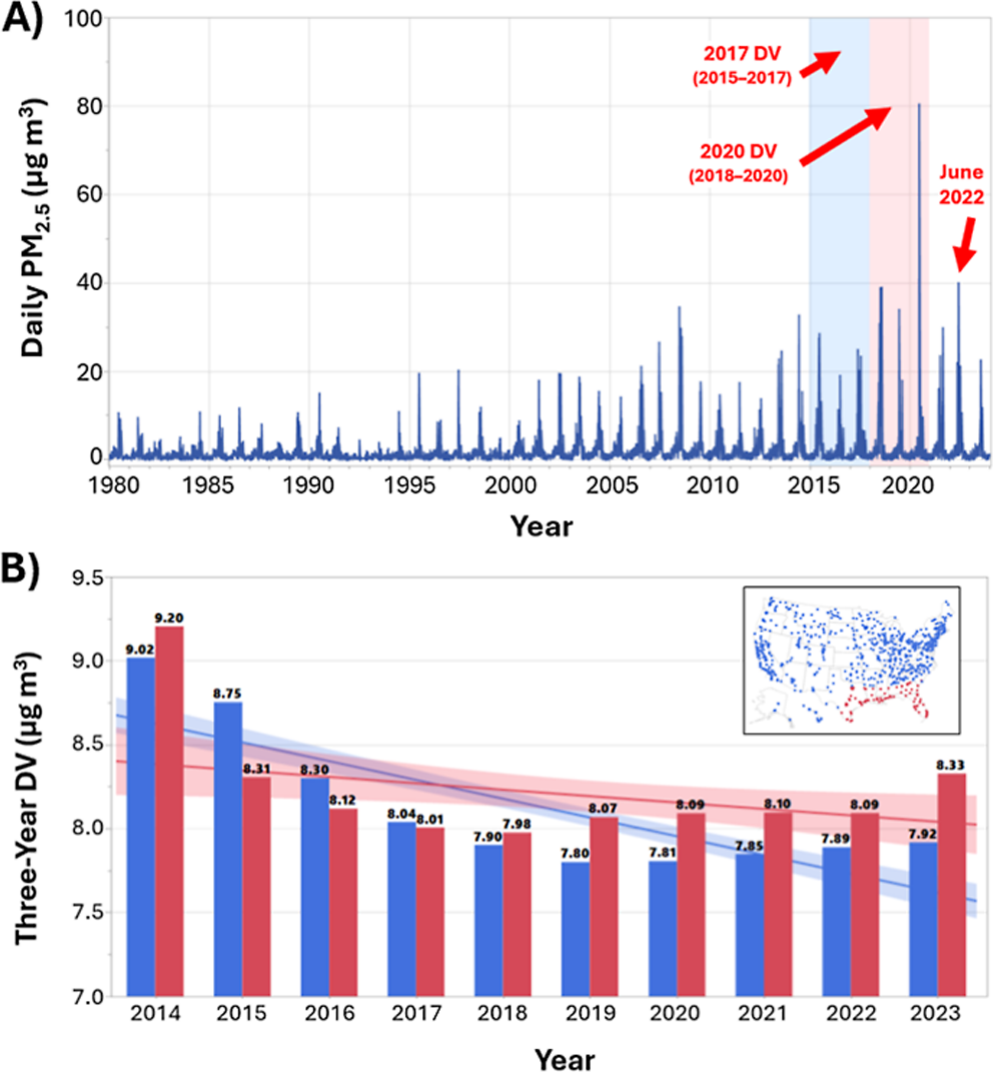
(A) Daily time series of MERRA-2 mean surface PM_2.5_ over the red bounding box (80–100°W; 23–33°N) marked in Figure 1a between 1980 and 2023. The June 2022 dust event is marked as well as the 2017 and 2020 DV periods. (B) Mean official EPA DVs (black labels; μg m^–3^) for all monitors within (red) versus outside (blue) the Figure 1a bounding box (see top-right inset) between 2014 and 2023. Like-colored linear trend lines and 95% confidence intervals are plotted for the respective groups in the background.

### 3.5 Contextualizing the June 2022 Dust Outbreak in Time, Space, and Environmental Policy

While the June 2022 Saharan dust outbreak is highlighted due to its coincidence with the EPA’s recent PM_2.5_ NAAQS revision, it is far from the only such event. Figure 5a displays the 44 year time series of daily dust PM_2.5_ averaged over the northern Gulf of America and neighboring Southeast U.S. coastal areas (see Figure 1a). The magnitude of the June 2022 dust event is not particularly striking compared to the seasonal dust activity in the 2010s. In particular, the June 2020 dust plume, termed the “Godzilla dust cloud” by popular media and researchers,^21,44,45^ yielded daily surface PM_2.5_ concentrations >35 μg m^–3^ across large portions of the Southeast U.S. (and locally >70 μg m^–3^).^46^ In addition to the 2020 Godzilla event, another strong dust intrusion occurred in 2018 (Figure 5a), both of which fell within the 2020 DV period (i.e., 2018–2020). While not necessarily causal, these dust episodes coincided with a 0.35 μg m^–3^ increase in the average DV for all Gulf-adjacent air monitors between 2017 and 2020 (Figure 5b). The mean 2017 DV for Gulf-adjacent monitors was 8.01 μg m^–3^ and nearly identical to the 8.04 μg m^–3^ average for all other monitors in the U.S. However, following the three years of elevated dust activity in the Gulf of America (Figure 5a), the 2020 DV increased to 8.09 μg m^–3^ among Gulf-adjacent monitors, whereas it declined to 7.81 μg m^–3^ elsewhere (Figure 5b).

## 4 Discussion

The June 2022 SAL incursion demonstrates the possibility of Saharan dust traversing the Atlantic Ocean and adversely impacting the Central U.S. air quality. While numerous monitor locations experienced nontrivial PM_2.5_ increases in both their annual mean and 2023 DVs, the potential regulatory relevance was greater along the western and central Gulf Coast. Though the June 2022 dust episode was a prominent example of westward Saharan dust migration, this is far from the only such instance (Figure 5a). In fact, the fingerprints of Saharan dust transport can be observed within the 25 year mean surface PM_2.5_ climatology from MERRA-2 (Figure 6). Whereas very little dust PM_2.5_ is observed over the Gulf of America in May (Figure 6a), a clear pathway of dust PM_2.5_ emerges during June, extending from the Caribbean and into the U.S. Gulf Coast (Figure 6b). In fact, the 5 μg m^–3^ contour captures the four air quality monitors in Table 1 during both June and July (Figure 6c), indicating that roughly 10% of the entire annual PM_2.5_ NAAQS is consumed by exogenous dust during these two months on average [i.e., (5 μg m^–3^ × 61 d)/(9 μg m^–3^ × 365 d) = 9.3%]. During July (Figure 6c), even the 10 μg m^–3^ contour expanded from the Caribbean Sea to enclose parts of South Texas. The dust PM_2.5_ eventually subsides climatologically during August with the 5 μg m^–3^ contour retreating to the far southern Gulf of America (Figure 6d).

**Figure 6:**
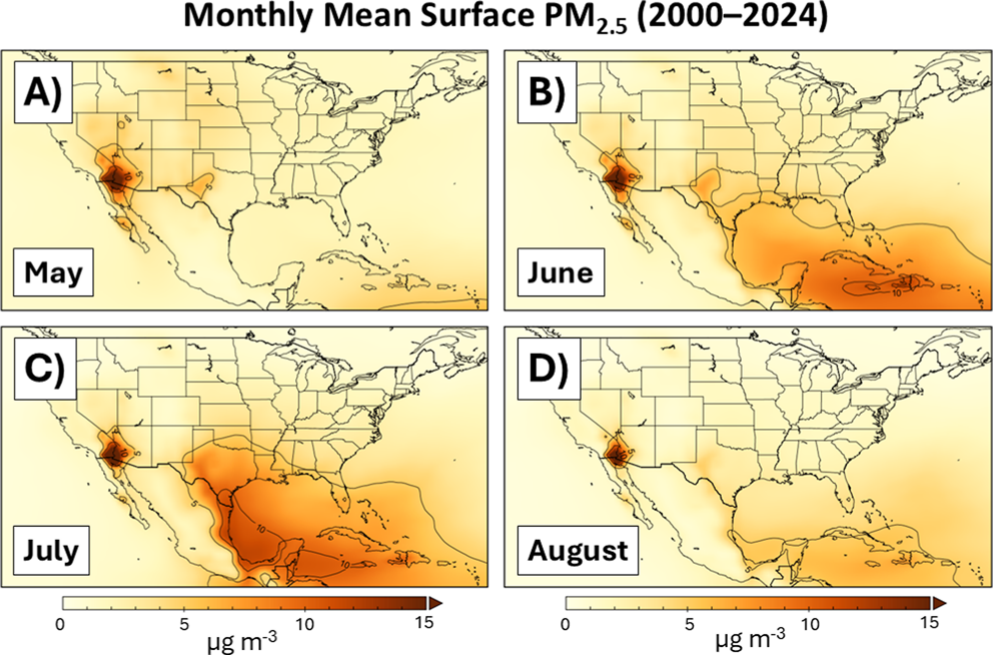
Monthly mean PM_2.5_ (μg m^–3^) during the most recent 25 years (2000–2024) according to the MERRA-2 reanalysis.

Though the June 2022 dust outbreak affected locations across the Central U.S., Figure 6 illustrates that the U.S. Gulf Coast is most susceptible to heavier dust loadings on an ongoing basis. While significant dust outbreaks are discrete events and do not appreciably occur every year, their regulatory significance, whenever they are observed, will be most poignantly experienced along the Texas and Louisiana coasts, a region with prominent manufacturing and refining industries. For instance, the Louisiana petrochemical sector is responsible for approximately 75,000 jobs and 14% of the state’s GDP.^47^ Meanwhile, in neighboring Texas, the national leader in chemical production, the chemical manufacturing industry accounts for $52.7 billion in state economic activity.^48^ Table S1 shows how these industrially intensive sectors in both states play outsized national roles. For instance, Louisiana’s contribution to the national petroleum manufacturing sector (8.82% of the U.S. total) is > 8× its overall contribution to the United States’ economy (1.13%). Similarly, Texas’s oil and gas extraction activities represent nearly half of the nation’s total (49.4%) and outweigh its overall economic footprint (9.32%) by a factor of 5 (Table S1). Commensurate with these economic activities, Figure S5 shows the reported PM_2.5_ emissions in both states relative to the continental U.S. according to data from the 2020 National Emissions Inventory (NEI).^49^ The industrial corridor between Houston, Texas, and New Orleans, La., where many of these manufacturing facilities are located, is readily apparent as a cluster of comparatively large PM_2.5_ emissions (though industrial activities contribute <15% of overall PM_2.5_ emissions in Louisiana^50^).

Taken in concert with Section 3, Figures 6 and S5 reveal how the same region containing a large fraction of the nation’s petroleum refining and chemical manufacturing capacity also experiences its greatest likelihood of Saharan dust outbreaks. Historically, significant Saharan dust outbreaks into the mainland U.S., such as those in 2015,[Bibr ref7] 2018, and 2020,^44,45^ did not create a regulatory dilemma because the NAAQS for annual PM_2.5_ was high enough (i.e., 12 μg m^–3^) that even strong Saharan dust episodes could not yield increases to the three-year DVs that would alter the attainment classification of the affected sites. However, with the tightening of the annual PM_2.5_ NAAQS in 2024, a larger number of air quality monitors nationwide now reside in the tipping-point range of Saharan dust (Figures 1a, 4d).

Thus, Saharan dust is unique among other natural aerosol-generating processes in that it is poised to represent a growing regulatory concern along the western and central Gulf Coast, as was the case for the 2023 DV at the Port Allen, La., monitor, as well as multiple other locations (Table 2). Within the revised regulatory landscape instituted in 2024, the intersection of heavy industrial infrastructure (Table S1 and Figure S5) and Saharan dust transport corridors over the U.S. Gulf Coast (Figure 6) will inevitably elevate the consideration of the SAL in regulatory discussions. This paper serves to highlight the new positionality of Saharan dust within the air resources policy space, in addition to its long-standing role as a climate modulator within the broader earth system.

## Supplementary Material



## Notes

Additional figures and tables detailing the synoptic-scale surface meteorological features associated with the trans-Atlantic Saharan dust outbreak, the aerosol composition of the plume, chronological evolution of the PM_2.5_, and spatial distribution of PM_2.5_ primary emissions (PDF)
