# Supplementary material for: The Expanded Regulatory Significance of Saharan Dust Plumes in the United States
Source: Environ Sci Technol. 2025 Sep 8;59(37):19745–54. doi: 10.1021/acs.est.5c02205 (PMC12461923; doi:10.1021/acs.est.5c02205)
Supplement: Supplementary file 1 [file es5c02205_si_001.pdf]

The Expanded Regulatory Significance of Saharan Dust Plumes in the United States

* Paul Miller[1, 2]

Kimberly Hamilton-Wims[3]

Ken Holmes[4]

Doug Melancon[4]

Jason Meyers[3]

Tegan Treadaway[4]

[1] Coastal Meteorology (COMET) Lab
Department of Oceanography and Coastal Sciences
Louisiana State University
93 S Quad Dr
Baton Rouge, LA 70803

[2] Coastal Studies Institute
Louisiana State University
93 S Quad Dr
Baton Rouge, LA 70803

[3] Office of Environmental Assessment
Louisiana Department of Environmental Quality
602 N 5th St
Baton Rouge, LA 70802

[4] Baton Rouge Complex
ExxonMobil
5955 Scenic Hwy
Baton Rouge, LA 70805

* Corresponding author: Paul Miller (pmiller1@lsu.edu)

Summary:

This article supplement consists of four pages (S1-S4) containing five graphics (Figures S1-S5), one table (Table S1), and references for the data they depict.

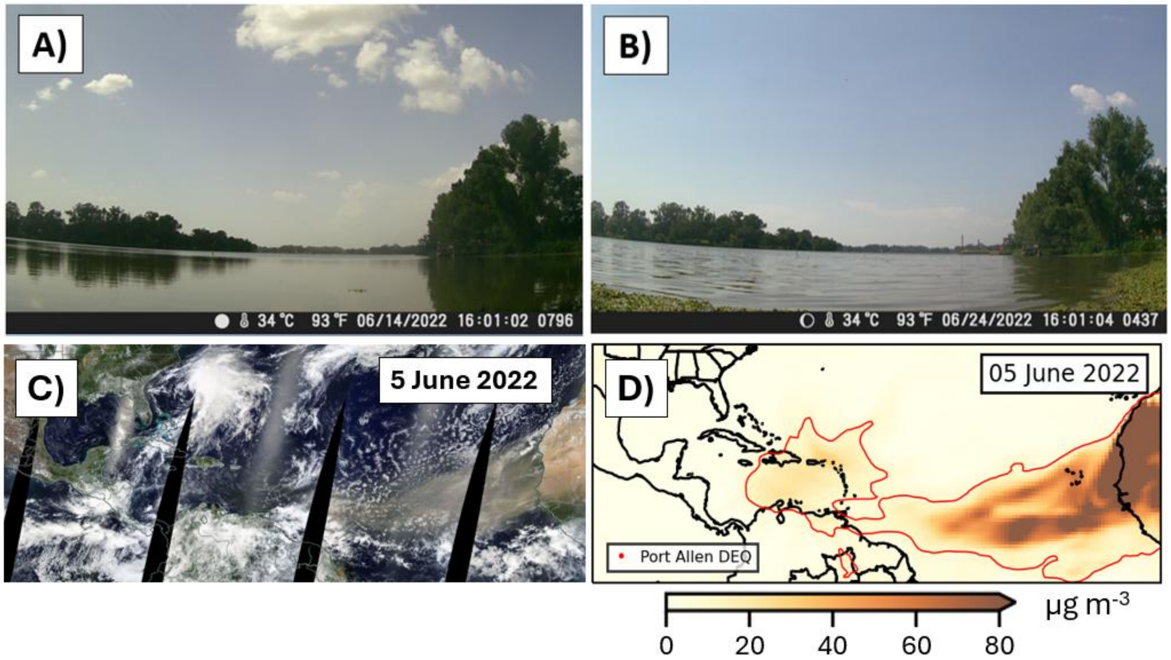

Figure S1. Saharan dust event as observed by a stationary trail camera in Baton Rouge, La., on (A) June 14 at approximately 2:00 PM LT, and (B) a comparable image taken ten days later at the same time during similar ambient sky cover and temperature. The difference in image hue is entirely due to the presence of high dust $PM_{2.5}$. (C) Terra MODIS true-color image from June 5, 2022. The nascent dust plume is visually identified as translucent brown plume of dust over the eastern Atlantic. (D) MERRA-2 dust-only $PM_{2.5}$ concentration for the 24-hour period beginning 0000 UTC on June 5, 2022. The Port Allen monitor is shown in a red circle, and the 9-µg m^{-3} contour is drawn in a red line.

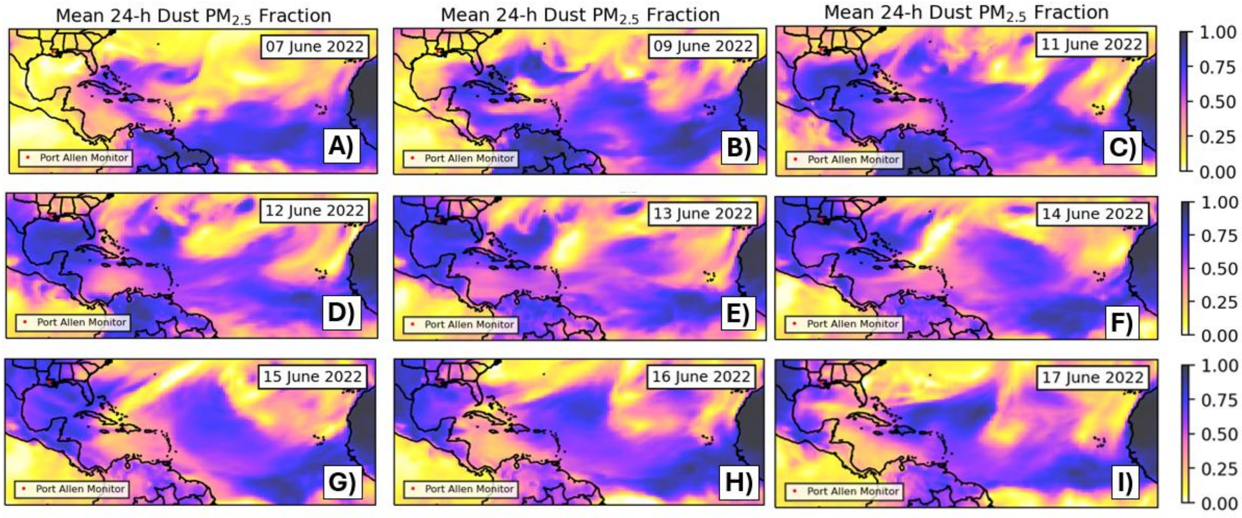

Figure S2. Same as Figure 2 in primary manuscript, but instead of the dust $PM_{2.5}$ mass concentration, the fraction of dust $PM_{2.5}$ to total dust $PM_{2.5}$ is shown. The plume is >75% dust, often larger as the plume approaches the Gulf Coast on 12-13 June 2022.

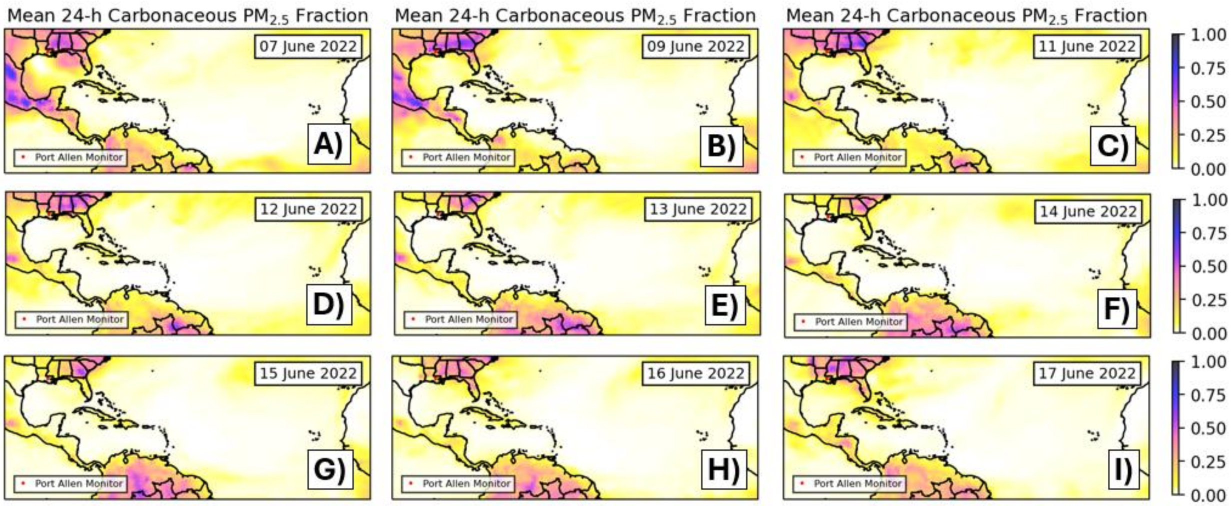

Figure S3. Same as Figure S2, but for the carbonaceous $PM_{2.5}$ fraction (organic carbon plus back carbon) indicative of biomass burnings[1].

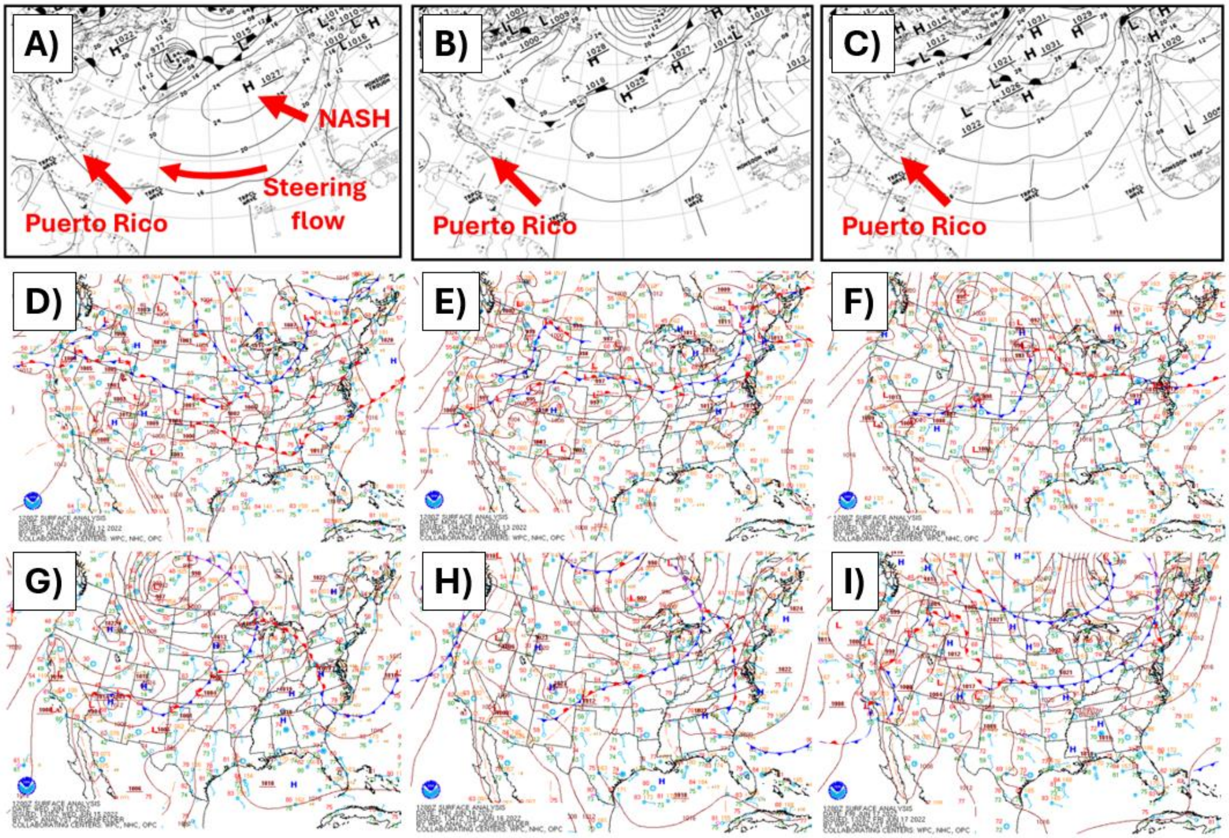

Figure S4. (A-C) Surface analysis of the north Atlantic Ocean from NOAA Ocean Prediction Center valid for 1200 UTC on June 7, 9, and 11, 2022. (D-I) A comparable surface analysis from the NOAA Weather Prediction Center of the continental U.S. for June 12–17, 2022, respectively.

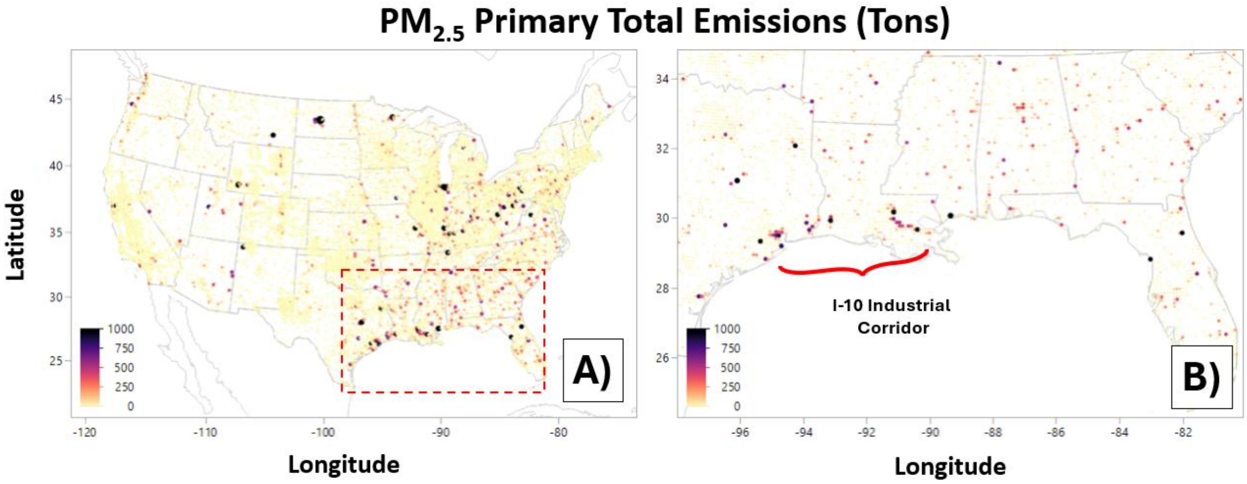

Figure S5. (A) Annual $PM_{2.5}$ primary emissions (short tons) according to the 2020 National Emissions inventory (NEI)[2]. Facility-level data were aggregated to ~10 km resolution for plotting purposes, and (B) an inset over the Gulf Coast. Reported $PM_{2.5}$ emissions are from the 2020 NEI NEI. The industrial corridor between Houston, Texas, and New Orleans, La., where many manufacturing facilities are located, is readily apparent as a cluster of comparatively large $PM_{2.5}$ emissions (though industrial activities contribute <15% of overall $PM_{2.5}$ emissions in Louisiana[3]).

Table S1. Texas and Louisiana respective share of overall national gross domestic product as well as sector-specific gross domestic product[4].

Activity	Louisiana	Texas
Total national gross domestic product	1.13%	9.32%
Petroleum and coal products manufacturing	8.82%	29.4%
Chemical manufacturing	4.25%	12.3%
Mining, quarrying, and oil and gas extraction	3.06%	49.4%

Supplementary references:

(1) Logan, T.; Dong, X.; Xi, B.; Zheng, X.; Wang, Y.; Wu, P.; Marlow, E.; Maddux, J. Quantifying Long-Term Seasonal and Regional Impacts of North American Fire Activity on Continental Boundary Layer Aerosols and Cloud Condensation Nuclei. *Earth and Space Science* **2020**, *7*, e2020EA001113. DOI: https://doi.org/10.1029/2020EA001113.

(2) EPA. *National Emissions Inventory*. 2020. https://www.epa.gov/air-emissions-inventories/2020-air-emissions-data (accessed 2025 February 13).

(3) EPA. *National and State EIS Sector CAPS Trends*. 2025. https://www.epa.gov/air-emissions-inventories/air-pollutant-emissions-trends-data (accessed 2025 February 13).

(4) U.S. Bureau of Economic Analysis. *SAGDP2N Gross domestic product (GDP) by state*. 2024. https://www.bea.gov/itable/regional-gdp-and-personal-income (accessed 2025 January 6).
